# Sexual and reproductive health services utilization and associated factors among secondary school students in Nekemte town, Ethiopia

**DOI:** 10.1186/s12978-018-0501-z

**Published:** 2018-04-17

**Authors:** Wakgari Binu, Taklu Marama, Mulusew Gerbaba, Melese Sinaga

**Affiliations:** 1 Department of Midwifery, Arba Minch College of Health Sciences, Arba Minch, Ethiopia; 2 Department of Midwifery, College of Health Sciences and Medicine, Wolaita Sodo University, Wolaita Sodo, Ethiopia; 3 Department of Population and Family Health, College of public Health and Medical Sciences, Jimma University, Jimma, Ethiopia

## Abstract

**Background:**

Despite policy actions and strategic efforts made to promote sexual and reproductive health service uptake of youths in Ethiopia, its utilization remains very low and little information was found on the extent to which school youths utilize available reproductive health services in Nekempt town. This study was aimed to assess utilization of Sexual and Reproduactive Health (SRH) services and its associated factors among secondary school students in Nekemte town, Ethiopia.

**Method:**

A school based cross-sectional study design was conducted from April 18 to 22, 2016. Multistage cluster sampling technique was used to select a total of 768 students who attended secondary schools. Sexual and reproductive health services utilization was measured using one item asking whether they had used either of sexual and reproductive health services components during the last one year or not. The data was entered using EpiData Manager with Entry Client and further analysis was done using SPSS version 21 software. Descriptive statistics, cross tabulations, biviarate and multivariate logistic regression analyses were used. All variables were set by *p*-values less than 0.05 and reported by Adjusted Odds Ratio with its 95%CI.

**Result:**

Out of the 768 study subjects, 739 participants underwent all the study components giving response rate of 96%. About 157 (21.2%) school youths reported that they utilized SRH services. On multivariable logistic regression analysis after adjusting for other variable, discussion with health workers (AOR 3.0, 95%CI [1.7–5.2]), previous history of perceived Sexually transmitted infections (STIs) symptoms (AOR 2.6, 95%CI [1.2–5.5]), being ever sexually experienced (AOR 5.9, 95%CI [3.4–10.2]) and exposure to information from school teachers (AOR 0.36, 95%CI [0.2–0.6]) were found to be independent determinants of sexual and reproductive services utilization among secondary school youths. Inconvenient times, lack of privacy, religion, culture, and parent prohibition were barriers to SRH service uptake cited by the school youths.

**Conclusions:**

The overall utilization of sexual and reproductive services was low among school youths in the town. Discussion with health workers, history of perceived STIs symptoms, sexual experience and information were the association factors of sexual and reproductive service utilization among secondary school youths.

## Plain English summary

Even if policy actions and strategic efforts made to promote sexual and reproductive health service uptake of youths in Ethiopia, few literatures showed its utilization remains very low. So, this study was aimed to assess utilization of Sexual and reproductive Health (SRH) services and its associated factors among secondary school students in Nekemte town, Ethiopia.

Students’ utilized as well as utilizing Sexual and reproductive Health services before and during April 18th to 22nd, 2016 are included in the study. Among 12 above grade 9 schools, two government and 2 non-government schools were selected using lottery methods. For this study, a total of 768 students were needed. To have these, students from each grade and sections were randomly selected from their attendance lists. On the day of data collection the randomly selected students were told to remain in their classes. Sexual and Reproductive Health services utilization was measured if the students utilized one of the six given components. To keep data quality first data was entered to EpiData Manager and exported to SPSS version 21 software to have the required output. The result was described in table and figures, and cross tabulated. Bivariate logistic regression model was used to see association between utilization and other factors.

In this study, only 157 (21.2%) of students reported their utilization of Sexual and Reproduction Health services. Students who discussed with health workers, perceived history of Sexually transmitted infections (STIs) symptoms previously, experienced sex ever and exposed to information from school teachers were found to be better utilized Sexual and Reproductive Health services.

When summarized, the overall utilization of SRH services was low among school youths in the town.

## Background

There were 1.8 billion young people aged 10–24 years among which 1.2 billion youth aged 15–24 years globally in 2015, accounting for one out of every six people worldwide in which Africa comprises 19% (above 226 million) of the global youth population [1]. Young people make up the greatest proportion of the population in sub-Saharan Africa, with more than one-third of the population 10–24 ages [2], and 33.8% of the total population in Ethiopia is between the age of 10–24 [3].

Since the International Conference on Population and Development Key Informant Interview held in Cairo in 1994, governments have pledged to improve the SRH of adolescents by providing access to comprehensive, appropriate information and education and youth friendly health services. Most regions of the world however, still fall short of these commitments, especially for unmarried young people [4].

Even though the comprehensive knowledge of HIV and other Reproductive Health (RH) problems is increasing around the world, many young people do not have the information or means to protect themselves from these problems [5]. Many health problems are contributed by adolescents and young people worldwide: 8.7 million abortions undergone, 41% of new HIV infection, high rate of early marriage and STIs, and high proportion of stillbirth and newborn deaths [6].

In Africa, 430,000 young people are infected with HIV per year; 2.6 million young people are living with HIV; teenage pregnancy rates still remain high and maternal mortality is among the leading causes of death for adolescent girls in this region [7]. Studies done in Ethiopia also show that there was 42.1% sexual risk behavior [8], 19% of youths reported having had premarital sexual intercourse with the mean age of 16.48 years at the first sexual intercourse [9], self-reported STIs prevalence was 19.5% [10] and abortion rate was also 65 per 1000 women [11].

Concerning the utilization of SRH services, it is not enough as expected from the efforts tried in Ethiopia. The SRH services utilization has great variation, 21.5% in Hadya [12] to 96.1%in Harar [13], that refers different factors are affecting the utilization differently in different part of the country. The SRH services utilization is high in areas where youth friendly services are available and accessible at community level [13]. It is evidenced that high school students were visiting SRH services to receive SRH information, for counseling service, to obtain a condom, for treatment of STI, for postabortion care [14] while others were not using SRH services because of inconvenience service hour, feel fear to be seen by others, too long waiting hours, providers are judgmental and unfriendly, feel embracement at seeking RH services [15]. Even, the utilizationof SRH services among secondary school students is not well explored in the country and the existing studies do not show enough information about the SRH situation of secondary school students, resulting in the absence of sustainable school based intervention for secondary chool students. So, additional study that assesses the magnitude and factors affecting SRH service utilization is very crucial to improve SRH service utilization of secondary school young people in the study area in the way that reduce morbidities and disabilities related to sexual and reproductive health.

## Methods

Institution based cross-sectional study design was conducted in Nekemte town, East Wallaga zone, Western Ethiopia from April 18th to 22nd, 2016. Nekemte Town is situated 331 km from Addis Ababa city. The town covers an area of 5480 ha. According to data from Central Statistics Agency Branch in the town, the total population of the town is projected to be 97,289 and young people in the town is estimated to be 37,796 (male = 19,626, female = 18,170) in 2016 [16].

According to the information from Nekemte Town Education Office, the total number of young people enrolled to secondary school (grade 9–12) in 2015/16 was 11,428 (male = 5539, female = 5867) students in seven government secondary schools 9771(85.5%) and five none-government (two Non-Governmental Organizations (NGO) and three Private) secondary schools 1657(14.5%). The government schools are free of charge for educational purposes. From the estimated total population of young people, only 30.24% (28.22% of male and 32.29% of female) of young people were attending schools. The data from Nekemte Town Health Office showed that the town has one general hospital, two government health centers, one higher and twelve medium private clinics, twelve NGO health facilities that are serving the society. From these health facilities, one government health centre and two NGO clinics were delivering SRH services and youth friendly services separately which is free of charge.

All secondary school students enrolled in the year 2015/2016 in the town were the source population and all the students in randomly selected secondary schools were the study population. Sample size was calculated using single population proportion formula by taking the proportion (p) of SRH services utilization by school youths to be 32% taken from the study conducted in Bahir Dar Town (19) [17] with the conservative assumptions in order to get enough sample size that would allow the study to look into various aspects of school youths. The assumptions of 95% confidence level (level of significance, z_α_ = 1.96), 5% margin of error, design effect of 2.0 and 15% non-response were used to determine the sample size. Accordingly, the total sample size was 768.

Concerning Sampling Techniques, Multistage cluster sampling technique was used in order to select a representative sample of students. Four schools (two from government ant two from non-government) were selected randomily. Samples were selected from government and non-government schools proportional to their size of the student population. The total sample was allocated to each grade from grade 9 to 12 proportionate to their student population size; Then four (2 government and 2 non-government) schools were selected using simple random sampling to recruit the allocated subjects for each grade. From each grade, sections were selected randomly and finally the study subjects were selected by using lottery method in SPSS using their attendance lists in the respective schools. On the day of data collection the randomly selected students were told to remain in their classes.

The dependent variable in this study was whether a participant had utilized SRH services within the last 12 months anywhere whether in government or private health institutions. This was measured through the dichotomous response (yes or no). The positive response was further validated with questions on the type of SRH services utilized. This included information and counseling on SRH issues, family planning, voluntary testing and counseling on HIV, abortion care, maternal and child care, testing and treatment of STIs. A positive (“yes”) response to any one of these services was regarded as service utilization. The questionnaire was developed by collecting and adopting after customizing into the study context from various literatures [18–20].

The quality of data were assured by translating questionnaires from English to Afan Oromo then back to English by another expert using properly designed and pretested questionnaire. The data was collected by two Diploma Nurses and supervised by 1 BSc Nurse who were trained for two days. The pretest was done on 5 % of the sample size in Gimbi secondary school, a 100 km Town from Nekemte and some modifications such as skipping pattern and sequence sections in the questionnaire were made. To maintain confidentiality, each participant took a single sparsely arranged seat and the participant put the questionnaire on separate table arranged at corner of the room.

The questionnaires were designed by using Epidata manger and entry-client. Regarding data analysis and management, all returned questionnaires were checked for completeness and consistency manually. Thereafter, data was coded, entered into EPI-data entry client 2.0.8.56 and exported to Statistical Package for the Social Sciences (SPSS) version 21. Frequencies and percentages were used to summarize descriptive statistics. Bivariate logistic regression analysis was done by entering variables that were found to affect SRH utilization. Variables with the *p*-value of less than or equal to 0.25 were entered into multivariate logistic regression. Those variables statistically significant at p-value less than 0.05 in multivariate logistic regression analysis were found to be taken as statistically significant. Adjusted odds ratio with the confidence level of 95% was considered to assess the strength of the association between dependent and independent variables.

Some phrases are Operationed as follows:

### Attitude

Respondents has favorable attitude if they score equal or above mean score (66.48) of the total 24 attitude questions with 1–5 likert scale points.

### Knowledge of SRH services

First knowledge about the SRH services was assessed by asking participants whether they were aware of SRH service components or not. Then SRH knowledge was assessed through 8-item scale on knowledge of SRH service components and the sum of scores ranging from one (minimum) to eight (maximum) for subjects were used in the analysis.

### SRH services utilization

This was measured through the dichotomous response (yes or no) by asking whether a participant had utilized one or more of SRH service components within the last 12 months. The positive response was further validated with questions on the type of SRH services utilized. A positive (“yes”) response to any one of these services was regarded as service utilization.

## Results

Out of the total of 768 study participants, 739 of students took part in the survey giving a response rate of 96.0%. Out of these, 349(47.2%) were males, 665 (90.0%) and 74(10.0%) falls under the age group of 15–19 and 20–24 years respectively with the mean age of 17.3(SD = ±1.7); 25(3.4%) and 136(18.4%) were single and in relationship respectively. Majority of them 925(97.4%) were Oromo by ethnic group, and protestant 525(55.5%) followers. Regarding their current educational status, h 82.9% and 17.1% of them were attending government and private schools respectively. The result also shows that the fathers 666(91.1%) and mothers 573(77.5%) of respondents were formally educated and 291(39.4%) of fathers and 220(29.8%) of mothers were employed. Majority of the students 486(65.8%) were living with their family (Table 1).

**Table 1:** Socio-demographic, community and family characteristics of secondary school students in Nekemte Town, Ethiopia, April 18–22, 2016

Socio-demographic characteristics		Frequencies	Percentage
Age	15–19	665	90.0
	20–24	74	10.0
Sex	Male	349	47.2
	Female	390	52.8
Marital status	Married	25	3.4
	In a love relationship	136	18.4
	Single	578	76.0
Ethnicity	Oromo	719	97.3
	Others	20	2.7
Religion	Protestant	405	54.8
	Orthodox	155	21.0
	Wakefata	140	18.9
	Muslim	28	3.8
	Others^***^	11	1.4
Educational status	Grade 9	297	40.2
	Grade 10	173	23.4
	Grade 11	134	18.1
	Grade 12	135	18.3
School type	Government	613	82.9
	Private	126	17.1
Pocket money in ETB	No money	224	30.3
	1–500	450	60.9
	> 500	65	8.8
Mother’s education	No formal education	166	22.5
	Formal education	573	77.5
Father’s education	No formal education	73	9.9
	Formal education	666	91.1
Mother’s occupation	Unemployed	519	70.2
	Employed	220	29.8
Father’s occupation	Unemployed	448	60.6
	Employed	291	39.4
Living arrangement	Living with family	486	65.8
	Not living with family	253	34.1
Family residence	Urban	534	72.3
	Rural	205	27.7

Others = Amhara, Guraghe, Tigrie Others^***^ = Adventist, Catholic, Jhoba

### Knowledge, attitudes towards SRH services and sexual practices

Three hundred fifteen (42.6%) and 30 (4.1%) of the respondents know at least one and eight SRH service components respectively. Six hundred eighty six of participants have an information on SRH services and 564 (76.3%) of participants were aware of at least one health facility where SRH services could be delivered. Government health facilities, private health facilities, NGO health facilities and traditional facilities were SRH services delivery points as cited by 509(68.9%), 286(38.7%), 187(25.3%) and 50(6.8%) of the respondents respectively. Majority of the respondents (72.7%) have involved in the available school clubs and 400 (54.3%) had discussed on SRH issues with friends followed by health workers 163 (22.1%). 387(52.4%) of respondents had favorable attitude towards the SRH services. A total of 140 (18.9%) of respondents had ever experienced sexual intercourse and 101 (13.7%) of respondents had sexual contact within the last 12 months. The mean age at first sexual contact of respondents was 15.14 (SD = ±2.98) years. This study also showed that 70 (69.3%) of the sexually active respondents were male and 16 (15.8%) of them had more than one sexual partners (Table 2).

**Table 2:** Knowledge, Attitudes towards SRH services and Sexual practices among secondary school youths in Nekemte Town, 2016

SRH services and sexual related		Frequencies	Percentage
Awareness of SRH services(*n* = 739)	Yes	686	92.8
	No	53	7.2
Awareness of Health facilities(*n* = 687)	Yes	564	76.3
	No	123	16.6
Source of information on SRH services (Multiple Response)	Parent talk	116	12.2
	Friends	198	26.8
	Relatives	60	8.1
	Health workers	271	36.7
	School teachers	305	41.3
	School clubs	149	20.2
	Medias	333	45.1
	Printed materials	154	20.8
Participated in school clubs	Yes	537	72.7
	No	202	27.3
Discussion on SRH topics with (Multiple Response)	Mother	88	11.3
	Father	32	4.3
	Brother/sister	66	9
	Friends	400	54.3
	Health workers	163	22.1
	Relatives	34	4.6
Awareness of health facilities where to get SRH services (Multiple Response)	Government	509	68.9
	Private	286	37.8
	Non-government	187	25.3
	Traditional	50	6.8
Sexually ever experienced	Yes	140	18.9
	No	599	81.1
Currently sexually active	Yes	101	13.7
	No	638	86.3
Currently sexually active	Male	70	69.3
	Female	31	29.7
Partners for sexually actives	Only one	85	84.2
	More than one	16	15.8
Attitude towards SRH services use(*n* = 737)	Unfavorable	350	47.5
	Favorable	387	52.5

**Fig. 1:**
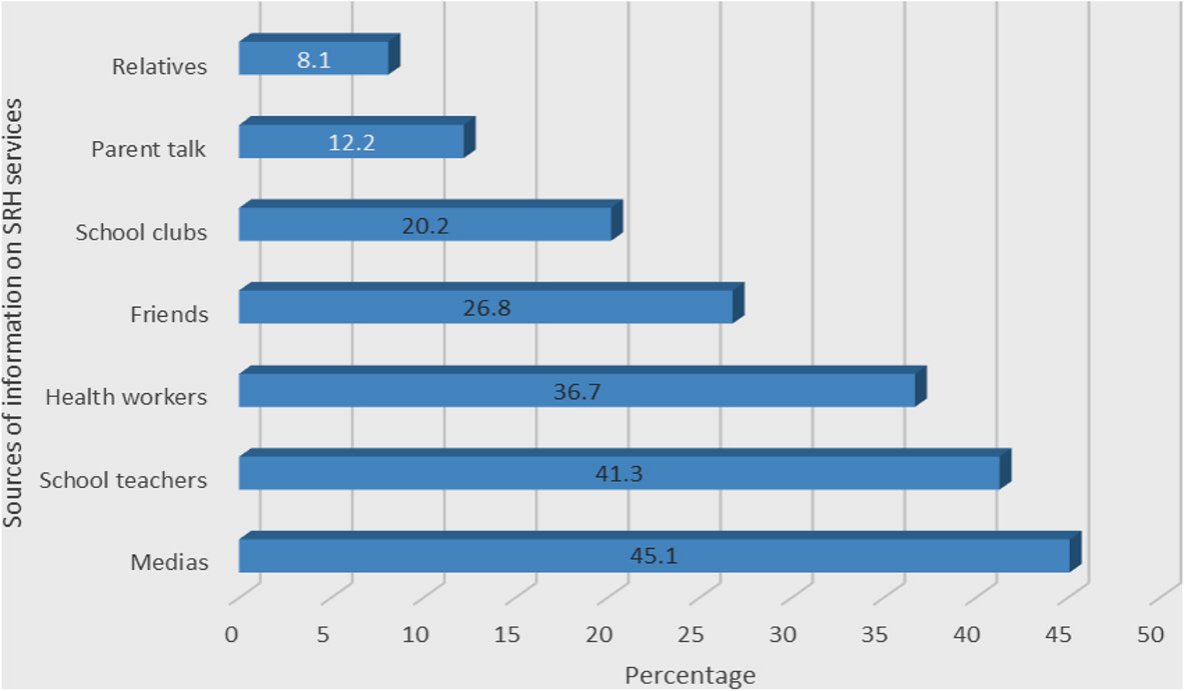
Sources of information on SRH services for secondary school youths in Nekemte Town, April 18-22, 2016

Respondents answered media 333(45.1%), teachers 305(41.3%), health workers 271(36.7%), friends 198(26.8%) as their potential sources of information for SRH services (Fig. 1).

### Utilization of SRH services

This study result showed that 157(21.2%) of overall study subjects had received at least one component of the SRH services in the last twelve months. Majority of the participants 116(54.8%) reported government health facilities from where they received SRH services followed by NGO health facilities 46(29.3%) (Table 3).

**Table 3:** SRH service utilization and reasons don’t use the services by secondary school youths in Nekemte Town, April 18–22, 2016

Characteristics		Frequency	Percentage
Utilized at least one SRH service (n = 739)	No	582	78.8
	Yes	157	21.2
SRH services utilized in the last 12 months (*n* = 157)			
Information and counseling on SRH issues		80	51
Family planning services		34	21.7
Pregnancy test		15	9.6
Pregnancy care		7	4.5
Abortion care services		5	3.2
Condom services		46	29.3
STIs treatment services		28	17.8
Voluntary testing and counseling for HIV		93	59.2
Sexually actives utilized FP methods (*n* = 101)	Yes	27	26.7
	No	74	74.3
Facilities where SRH services received (n = 157)			
Government		86	54.8
Private		35	22.3
Non-government		46	29.3
Traditional		6	3.8

N.B. System missed values were excluded from analysis, some observations may exceed 100% due to multiple options and others may be less than 100% due to missed values

**Fig. 2:**
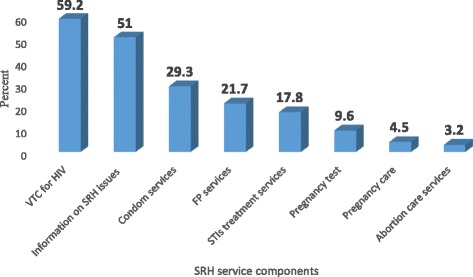
Components of SRH services utilized by secondary school youths in Nekemte Town, 2016

The most frequently utilized SRH services was Volunteer Test and Counseling 93(59.2%) followed by information and counseling on SRH issues 80(51%) and condom service 46(29.3%). The result also showed 27(26.7) of the sexually active respondents had utilized family planning methods in the last twelve month (Fig. 2).

**Fig. 3:**
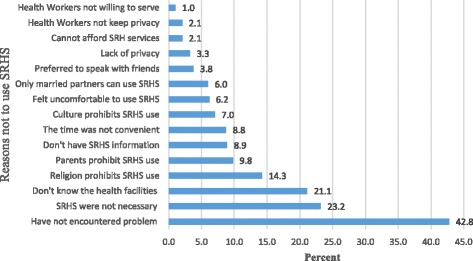
Reasons not to utilize SRHS by secondary cchool youths in Nekemte Town, 2016

In this study, 582(78.8%) of the study participants did not use SRH services in the last twelve months. The most frequently reported reasons for not utilizing SRH services, are not encountering any problem 249(42.8%),and believing that the services were not necessary 135 (23.2%) majorly (Fig. 3).

### Factors associated with utilization of SRH services

During bivariate analysis, being in 20–24 age (COR 2.36, 95%CI [1.34 to 4.16]), being married (COR 2.69, 95%CI [1.14 to 6.34]), history STIs (COR 4.41, 95%CI [2.46 to 7.91]), having ever sexual contact (COR 7.51, 95%CI [4.87 to 11.6]), and having favorable attitude towards SRH services (COR 1.46, 95%CI [1.01 to 2.13]), heard SRH information from school teachers (COR 0.4, 95%CI [0.27, 0.59]) discussing on SRH issues with friends (COR 0.46, 95%CI [0.3, 0.72]), discussing on SRH issues with health care workers (COR 2.78, 95%CI [1.8, 4.3]) and attending non-government schools (COR 0.46, 95%CI [0.27–0.79]) were found to be associated with SRH services utilization (Table 4).

**Table 4:** Bivaraite and Multivariate logistic regression analysis of factors associated with SRH services utilization among secondary school students, Nekemte, April 18–22, 2016

Variables		SRH service utilization		COR (95%CI)	COR (95%CI)
		Yes (%)	No (%)		
Age					
15–19		132(84.1)	374(92.6)	1	1
20–24		25(15.9)	30(7.4)	2.36 (1.34–4.16)*	1.15(0.51-2.57)
Marital status					
Married		11(7.0)	11(2.7)	2.69(1.14–6.34)*	1.9(0.58-6.48)
Single		146(93.0)	393(97.3)	1	1
School type					
Government		139(88.5)	315(78.0)	1	1
Non-government		18(11.5)	89(220)	0.46(0.27–0.79)*	0.56(0.26-1.20)
Discussion on SRH					
With friends	Yes	76(60.3)	239(76.6)	0.46(0.3–0.72)*	0.73(0.39-1.37)
	No	50(39.7)	73(23.4)	1	1
With health workers	Yes	59(46.8)	75(24.0)	2.78(1.8–4.3)*	3(1.72-5.24)^***^
	No	67(53.2)	237(76.0)	1	1
Sources of SRH services					
School teachers	Yes	47(29.9)	209(51.7)	0.4(0.27–0.59)*	0.36(0.21-0.61)^***^
	No	110(70.1)	195(48.3)	1	1
History of STIs	Yes	33(24.3)	22(6.8)	4.41(2.46–7.91)*	2.61(1.24-5.49)^**^
	No	103(75.7)	303(93.2)	1	1
Sexually ever experienced	Yes	79(50.3)	48(11.9)	7.51(4.87–11.6)*	5.87(3.38-10.19)^***^
	No	78(49.7)	356(88.1)	1	1
Attitude for SRH services					
Unfavorable attitude		62(39.7)	198(49.1)	1	1
Favorable attitude		94(60.3)	205(50.9)	1.46(1.01–2.13)*	1.21(0.69-2.10)

*significant at bivatiat e ^**^ significant at multivariate, *p* < 0.05 ^***^ significant at multivariate, *p* < 0.001

After controlling the effects of confounding, multivariate logistic regression analysis showed that the likelihood of SRH service utilization among secondary school was about 3 times (AOR = 3, 95%CI [1.72 to 5.24]) higher among respondents who discussed SRH issues with health workers than their counterparts. Students who had heard SRH issues from their school teachers were 64% (AOR = 0.36, 95%CI [0.21 to 0.61]) less likely to utilize SRHS than those who had not heard SRH related issues from their school teacher. The odds of SRH service utilization was about 6 times [AOR = 5.87, 95%CI [3.38 to 10.19)] higher among respondents who ever had sexual contact than their counterparts. Respondents who had history of perceived STIs symptoms were 2.6 times (AOR 2.61, 95%CI [1.24 to 5.49]) more likely to utilize the SRH services.

## Discussion

The overall utilization of SRH among school youths was 21.2% (95% CI[18.3–24.0]). It is similar with the cross-sectional study conducted in Mekele Town and East Gojjam zone [21, 22]. However, this finding was less than findings from Harar Town, Bahir Dar Town and Hadiya zone [12, 15, 23]. The possible reason for the discrepancy might be due to respondent characteristics, socio-demographic backgrounds and time reference used in the definition of SRH service utilization [24].

Around 13.7% of the students (9.5% male) were found to be sexually active within the last 12 month. Even though it seems low percentage of respondents, sexual behavior may have been masked because of the socio-cultural context, where sexual intercourse out of marriage is taboo.This result was consistent with another study from Mekele and Nepali [22, 25]. But this percentage is lower than studies in Bahir Dar and Hadiya [17, 26]. The difference might be due to socio-cultural background in which early marriage is encouraged in the two areas. In addition, the number of sexually active male students is greater than of female student. This might indicate that male are having sex with out of school females. The mean age at first sexual contact of respondents was 15.14 indicating early initiation of sexual intercourse. Sixteen (15.8%) of sexually active respondents had multiple sexual partiner that may pose them for risky sexual behavior and these are important indicators of exposure to risk of pregnancy and STI during adolescence. This finding was in line with the study conducted in Mekele Town [22].

Majority of sexually active respondents 62(61.4%) had received at least one component of the SRH services in the last twelve months. This finding goes in line with the finding from Hadiya zone [14]. The possible justification of this may be sexually active respondents were more exposed for SRH problems so that they were concerned about their sexual and reproductive healths than sexually inactive respondents.

The commonly utilized SRH service component was voluntary testing and counseling service (59.2%) which is almost similar with the findings of studies done in Goba Town and Gondar Town [27, 28]. Information and counseling on SRH issues, condom services and STIs services were also commonly recieved by 51.0%, 29.3% and 17.8% of the SRH services seekers respectively. This finding somewhat goes in line with the finding in Mekele Town [22]. Family planning service was utilized by 26.7% of the sexually active respondents which was similar to the findig from Nepali [25, 29] but less than findings in Gondar Town and Goba Town [27, 28]. The possible explanation for this difference might be due to more implimentation opportunities at the community than at school level and information gap among school youths of the study area as more government and non-government facilities are designed and implementing at community level.

The findings of this study indicated that school youths used various health facilities in which government health facilities most frequently cited (50.9%) followed by NGO health facilities similar to those previous studies conducted in Bahir Dar [17]. The possible explanation for this could be the services in governmental and NGO health institutions were given either free of charge or with a minimal payment.

This study also showed that a small proportion (3.8%) of the participants used traditional health service that goes in line with Bahir Dar and East Gojjam study [17, 21]. Even if this proportion seems insignificant, it needs a special concern because unless it is confirmed by health experts traditional treatments may pose health risks to individuals.

For the successfulness of the YSRH program appropriate and relevant information about SRH should be delivered to youths. In this study, Medias (45.1%), teachers (41.3%) and health workers (36.7%) were found to be the main source of information about SRH services which was in line with the finding from Mekele [22].

The result of this study showed that majority of the respondents had not utilized SRH services diferrent reasons. Absence of SRH problems for a moment and the service was not necessary for the moment were most commonly cited reasons not to use SRH services. Religion, cultural and parent prohibition, inconvenience service hour and lack of privacy at delivery point were also barriers to SRH services utilization. This finding was consistent with the findings of East Gojjam, Bahir Dar and Mtwara Tanzania [17, 21, 30]. This implies that there is a need of tackling the barriers by dealing with the community leaders, religious leaders, families and health service systems.

In this study, discussion with health workers, previous history of perceived STIs symptoms, being ever sexually experienced and exposure to information from school teachers were found to be independent determinants of SRH services utilization among school youths.

This study revealed that discussion of the service with health care workers had a significant association with SRH service utilization. This can be justified by the fact that discussion of services with people allows youths to create more opportunities to exchange information, experiences, and build comprehensive knowledge about SRH. It can also create opportunities to deal with adolescent problems associated with SRH service utilization so that health professionals might be the source of accurate information for youths which help them for appropriate decision making in health services seeking behavior.

School youths who had encountered at least one of STIs symptom were more likely to utilize SRH services than who did not encounter the problem. And also individuals who had ever sexual contact were more likely to utilize SRH services than abstainers. This finding goes in line with study conducted in Hadiya, Nepali and Bahir Dar [12, 15, 29] This might be explained as youths were more concerned about their health when they encounter SRH problems that triggered them to use SRH services and youths who engaged in sexual intercourse were more vulnerable to SRH problems that might increase the need for SRH services utilization. This implies that youths need an access to a wide range of health information and services as well as health professional support to engage in healthy and safe behavior.

The students who had heard SRH related issues from their school teachers seemed to be 64% less likely to utilize SRH service than students who had not heard from school teachers in contrary to the fact that school teachers were the main sources of information for SRH. The justification of this finding is not simple and difficult to hypothesize that information leads to less SRH service utilization. In fact school teachers may strictly disseminate information that early sexual initiation can lead to risk of unwanted pregnancy and STIs. Therefore, before formulating hypotheses about information from school teachers, additional studies using qualitative designs are needed to dig out the deeper meaning and to identify type of information delivered by school teachers.

### Limitation

Since this study examines personal and sensitive issues, obtaining honest responses among adolescent students might have been difficult. Therefore this data might have prone to respondent bias. The quantitative study design did not allow for probing into certain areas which needed further qualitative description. Finally, the study was conducted in schools of only one town, which means the findings may not be generalizable to the overall Ethiopian adolescent and youth population, who are socio-economically, linguistically, and ethnically diverse. In addition factors at community (e.g. parental attitude and control over the children) and health system are needed to be included in the future researches.

## Conclusion

This study had showed that low proportion of the school youths visited different health facilities to utilize SRH services in the last 12 months. The most frequently utilized SRH service component was voluntary testing and counseling service followed by information and counseling on SRH issues. Media was the potential source of information on SRH services for school youths followed by teachers and health workers. Discussion with health workers, previous history of perceived STIs symptoms, being ever sexually experienced and exposure to information from school teachers were found to be independent determinants of SRH services utilization among school youths.

## Abbreviations

NGOs: Non-Governmental Organizations
RH: Reproductive Health
SPSS: Statistical Package for the Social Sciences
SRH: Sexual and Reproductive Health
SRHS: Sexual and Reproductive Health Services
